# Strengthening the Role of *PSMC5* as a Potential Gene Associated with Neurodevelopmental Disorders

**DOI:** 10.3390/ijms26136386

**Published:** 2025-07-02

**Authors:** Mirella Vinci, Antonino Musumeci, Carla Papa, Alda Ragalmuto, Salvatore Saccone, Concetta Federico, Donatella Greco, Vittoria Greco, Francesco Calì, Simone Treccarichi

**Affiliations:** 1 Oasi Research Institute-IRCCS, 94018 Troina, Italy; mvinci@oasi.en.it (M.V.); amusumeci@oasi.en.it (A.M.); cpapa@oasi.en.it (C.P.); aragalmuto@oasi.en.it (A.R.); dgreco@oasi.en.it (D.G.); vgreco@oasi.en.it (V.G.); streccarichi@oasi.en.it (S.T.); 2 Department Biological, Geological and Environmental Sciences, University of Catania, Via Androne 81, 95124 Catania, Italy; salvatore.saccone@unict.it (S.S.); concetta.federico@unict.it (C.F.)

**Keywords:** 26S proteasome, the 20S regulatory subunit, ubiquitin proteasome system

## Abstract

The 26S proteasome is a large, ATP-dependent proteolytic complex responsible for degrading ubiquitinated proteins in eukaryotic cells. It plays a crucial role in maintaining cellular protein homeostasis by selectively eliminating misfolded, damaged, or regulatory proteins marked for degradation. In this study, whole-exome sequencing (WES) was performed on an individual presenting with developmental delay and mild intellectual disability, as well as on both of his unaffected parents. This analysis identified a de novo variant, c.959C>G (p.Pro320Arg), in the *PSMC5* gene. As predicted, this gene shows a very likely autosomal dominant inheritance pattern. Notably, *PSMC5* has not previously been associated with any phenotype in the OMIM database. This variant was recently submitted to the ClinVar database as a variant of uncertain significance (VUS) and remains absent in both gnomAD and dbSNP. Notably, it has been identified in six unrelated individuals presenting with clinical features comparable to those observed in the patient described in this study. Multiple in silico prediction tools classified the variant as pathogenic, and a PhyloP conservation score supports strong evolutionary conservation of the mutated nucleotide. Protein structure predictions using the AlphaFold3 algorithm revealed notable structural differences between the mutant and wild-type PSMC5 proteins. We hypothesize that the p.Pro320Arg substitution alters the structure and function of PSMC5 as a regulatory subunit of the 26S proteasome, potentially impairing the stability and activity of the entire complex. Although functional studies are imperative, this study contributes to a deeper understanding of *PSMC5*, expands the spectrum of associated neurodevelopmental phenotypes, and highlights its potential as a therapeutic target. Furthermore, this study resulted in the submission of the identified variant to the ClinVar database (SCV006083352), where it was classified as pathogenic.

## 1. Introduction

The 26S proteasome is a large, ATP-dependent proteolytic machine complex responsible for degrading ubiquitinated proteins in eukaryotic cells. It plays a critical role in maintaining cellular protein homeostasis by selectively removing misfolded, damaged, or regulatory proteins tagged with ubiquitin [1]. The 26S proteasome consists of a 20S catalytic core particle and one or two 19S regulatory particles that recognize and unfold ubiquitinated substrates [2,3]. This tightly regulated degradation process is essential for numerous cellular functions, including protein homeostasis, DNA synthesis, transcription, translation, cell cycle control, signal transduction, and stress responses [4]. The 26S proteasome is a multicatalytic proteinase complex with a highly ordered structure composed of two complexes, a 20S core and a 19S regulator. The 20S core is composed of four rings of twenty-eight nonidentical subunits; two rings are composed of seven alpha subunits, and two rings are composed of seven beta subunits. The 19S regulator is composed of a base, which contains 6 ATPase subunits and 2 non-ATPase subunits, and a lid, which contains up to 10 non-ATPase subunits. Proteasomes are distributed throughout eukaryotic cells at high concentrations and cleave peptides in an ATP/ubiquitin-dependent process via a nonlysosomal pathway. An essential function of a modified proteasome, the immunoproteasome, is the processing of class I MHC peptides.

Recent advances in high-throughput sequencing have facilitated the identification of novel candidate genes associated with neurodevelopmental disorders. Among these, neurodevelopmental proteasomopathies have emerged as a complex group of syndromes caused by defects in genes related to key proteasome systems, such as the ubiquitin–proteasome system (UPS) and the previously mentioned 26S proteasome [5,6]. These syndromes are typically characterized by delayed psychomotor development, behavioral abnormalities, facial dysmorphisms, and multisystemic anomalies.

The proteasome 26S subunit ATPase 5 (*PSMC5*) has emerged as a potential candidate for these diseases. Although its MIM phenotype number is not associated with a specific phenotype, it was previously associated with neurodevelopmental disorders [7]. This gene encodes proteasome regulatory subunit 8, which is pivotal for the formation of the 19S core subunit. PSMC5 is an ATPase that belongs to the AAAs (ATPases associated with diverse cellular activities) family, known for its nonproteolytic chaperone-like functions [8,9]. In addition to participating in proteasome functions, this subunit may participate in transcriptional regulation since it has been shown to interact with the thyroid hormone receptor and retinoid X receptor-alpha.

In addition to its role in proteasome-mediated protein degradation, this subunit may also be involved in transcriptional regulation, as it has been shown to interact with both the thyroid hormone receptor and retinoid X receptor-alpha [10]. As documented, the intracerebroventricular administration of shRNA PSMC5 in mice resulted in neuroprotective effects. In fact, PSMC5 was identified as a potential therapeutic target for the treatment of neurodegenerative diseases involving neuroinflammation-associated cognitive deficits and motor impairments induced by microglial activation [11].

In this study, whole-exome sequencing (WES) identified a de novo variant, c.959C>G (p.Pro320Arg), in the *PSMC5* gene in a patient presenting with global developmental delay and mild intellectual disability. We propose this variant as a potential mutational hotspot, as it has previously been identified in six individuals with clinical features similar to those observed in our patient. This study aims to strengthen the association between *PSMC5* and neurodevelopmental disorders, corroborating evidence reported in previous research.

## 2. Results

### 2.1. Clinical Report

The patient was a female second-born child, delivered at term following a pregnancy complicated by a diagnosis of Hashimoto’s thyroiditis at the fifth month of gestation. The patient was treated with levothyroxine (Eutirox). Delivery was unremarkable (eutocic). Psychomotor development was delayed. Previous genetic investigations performed at other centers included conventional karyotyping, array CGH, and methylation testing for Angelman Syndrome (AS), all of which returned normal results.

In 2015, the patient was hospitalized at Oasi IRCCS Research Institute and was discharged with a diagnosis of mixed specific developmental disorder. In 2023, she was readmitted for follow-up and further diagnostic investigations due to emerging behavioral disturbances.

Phenotypic evaluation revealed a body weight of 49 kg and a height of 154 cm. The patient exhibited muscular hypotonia and distinct facial dysmorphisms, including a small mouth, slightly down-slanting palpebral fissures, and a nasal tip mildly turning downward. Additional clinical findings included a pilonidal sinus, valgus knees, and bilateral flat-valgus pronated feet. A thyroid goiter was also noted on physical examination. Thyroid ultrasound revealed a significantly enlarged left lobe with a hypoechoic and heterogeneous appearance, resembling a pseudonodular pattern. The liver appears within normal limits, with regular contours and preserved echotexture. The gallbladder is in its normal position, with regular walls and no echogenic images suggestive of lithiasis within its lumen. The intrahepatic and extrahepatic biliary tracts, as well as the portal vein, have a normal appearance. The pancreas is normal in size and morphology, with preserved echotexture. The spleen is within normal limits, with regular contours. The kidneys are in their usual position, with a normal shape and volume, appropriate parenchymal representation, and a normal corticomedullary ratio, with no evidence of lithiasis. The urinary bladder has a regular wall thickness.

Over the past year, oppositional behavior, mood swings, and episodes of aggression have been reported (e.g., throwing objects, punching doors, and occasionally showing physical aggression toward her mother, siblings, and grandmother). These behavioral issues are not observed in the school environment or other social contexts.

Limited willingness to engage in interaction, with frequent avoidance and withdrawal behaviors (e.g., covering her face, turning away, becoming mute), as well as oppositional traits, has also been reported. At discharge, she received a diagnosis of syndrome of global developmental delay (HP:0001263) with mild intellectual disability (HP:0001256).

### 2.2. Next-Generation Sequencing (NGS)

**Figure 1:**
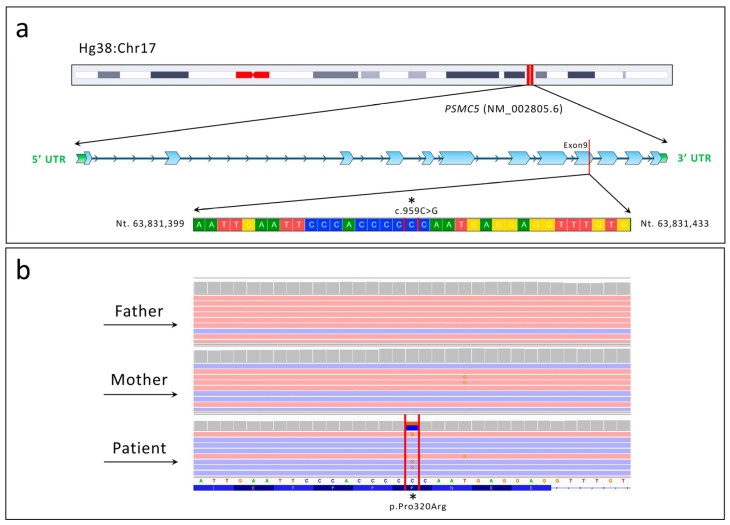
Chromosomal localization and next-generation sequencing (NGS) analysis identifying the c.959C>G (p.Pro320Arg) variant in the *PSMC5* gene (NM_002805.6). (**a**) Schematic representation of the chromosomal localization of the *PSMC5* gene, highlighting the identified variant within exon 9. (**b**) Integrative Genomics Viewer (IGV) snapshot showing the heterozygous c.959C>G variant in the patient, absent in both healthy parents, confirming its de novo origin.

WES analysis identified the de novo variant c.959C>G p.Pro320Arg within the *PSMC5* (NM_002805.6) gene (Figure 1).

The DOMINO tool indicated a very high probability of autosomal dominant (AD) transmission, with a score of 0.981 (where 0 indicates autosomal recessive inheritance and 1 indicates autosomal dominant inheritance). The variant was confirmed via conventional Sanger sequencing.

According to the ACMG criteria, the variant was classified as pathogenic. The PhyloP conservation score was 7.905, indicating strong conservation of the mutated nucleotide. Furthermore, multiple in silico algorithms classified the genetic variant as strongly pathogenic (Table 1).

### 2.3. Protein Structural Prediction

**Figure 2:**
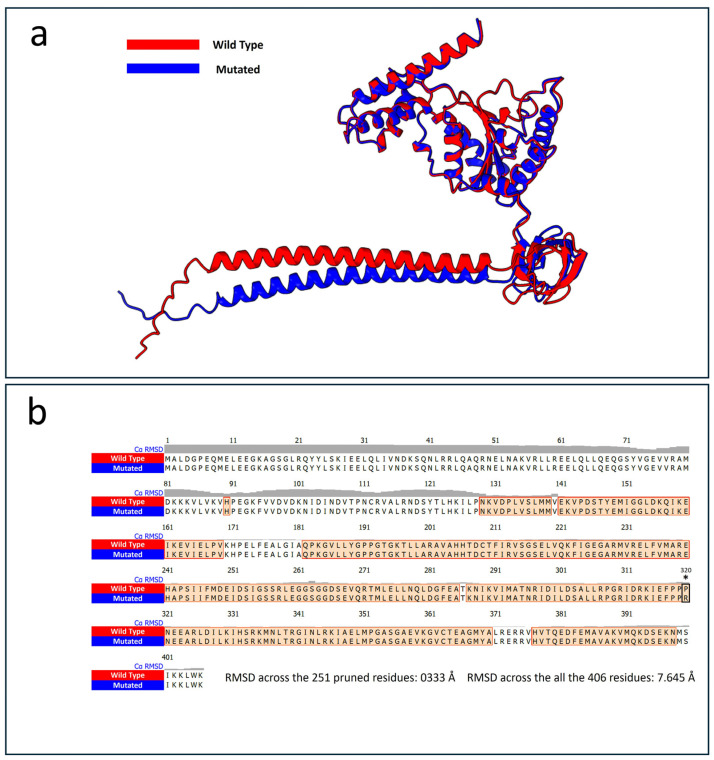
Protein structure alignment performed with the two best predicted models of both the wild-type (red) and the mutated Pro320Arg PSMC5 proteins (blue). (**a**) Graphical representation of the superimposed structure alignment carried out using the best AlphaFold3 models for both the wild-type and the mutated Pro320Arg PSMC5. Superimposition was carried out using UCSF ChimeraX software version 1.8. (**b**) Depiction of the aligned amino acids resulting from the protein structure alignment. The root main standard deviation (RMSD) is indicated in the figure and is expressed in Angstroms (Å). Asterisk indicates the specific mutation site.

Protein structure prediction analysis using the AlphaFold 3 algorithm was performed, selecting the best model based on the highest predicted local distance difference test (plDDT) score. The comparison between the wild-type and mutated PSMC5 structures revealed notable differences. Protein alignment showed that 251 out of 406 residues were aligned with a root-mean-square deviation (RMSD) of 0.333 Å, indicating close structural similarity in that region (Figure 2).

However, when considering all 406 residues, the RMSD increased to 7.645 Å, reflecting overall structural deviation. Despite the apparent similarity from the aligned region, alterations were observed in the hydrogen bond patterns and overall organization of the mutated protein compared to the wild type. The total number of hydrogen bonds observed in the selected model was 339 for the wild-type PSMC5, compared to 337 in the mutated protein. Table 2 lists the 23 unique hydrogen bonds present only in the wild-type protein and absent in the mutated p.Pro320Arg.

As predicted, the mutated PSMC5 protein exhibited a total of 21 hydrogen bonds that were not present in the wild-type structure. These unique interactions are listed in Table 3.

**Figure 3:**
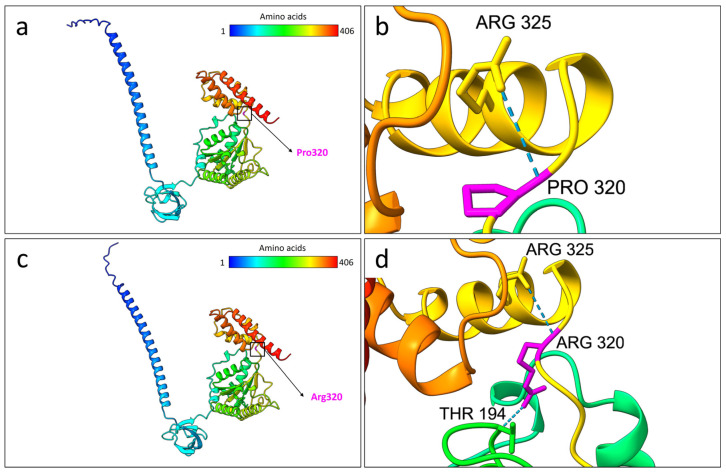
Structural comparison between the selected best models of the wild-type and Pro320Arg-mutated PSMC5 proteins based on AlphaFold3 predictions. (**a**) Predicted 3D model of the wild-type PSMC5 protein. The color gradient represents the amino acid sequence from the N-terminus (blue) to the C-terminus (red). The position of Pro320 is highlighted by both a black square and an arrow. (**b**) Close-up view of the wild-type Pro320 residue (magenta), showing a hydrogen bond with Arg325. (**c**) Predicted 3D model of the Pro320Arg-mutated PSMC5 protein, colored from the N-terminus (blue) to the C-terminus (red). The mutated Arg320 residue is indicated by a black square and arrow. (**d**) Close-up view of Arg320 (magenta) in the mutant protein, forming two hydrogen bonds with Arg325 and Thr194. All the protein models were generated using the AlphaFold3 server and visualized using UCSF ChimeraX version 1.8.

As predicted by the structural prediction analysis, the wild-type residue Pro320 formed a hydrogen bond with Arg325. On the other hand, the mutated Arg320 formed an additional bond with Thr194. These bonding differences in the AlphaFold3 models are depicted in Figure 3.

## 3. Discussion

In this study, we report an individual presenting with global developmental delay and mild intellectual disability. WES identified the de novo genetic variant c.959C>G p.Pro320Arg in the *PSMC5* gene. NGS analysis did not reveal pathogenic variants in known genes associated with the patient’s phenotype. Additionally, conventional karyotyping, array CGH, and methylation testing for Angelman Syndrome (AS) yielded normal results. However, we cannot rule out the possibility that other genetic variants not detected by WES may have contributed to the patient’s clinical presentation.

As reported by the DOMINO tool, PSMC5 shows an autosomal dominant inheritance pattern. Currently, there are no MIM phenotype numbers associated with *PSMC5* with a specific phenotype. Notably, the PSMC5 Foundation (https://psmc5.org/) (accessed on 23 May 2025) was established to support research into diseases with neurodevelopmental features associated with the PSMC5 gene. The foundation was created owing to the efforts of three families, with the aim of advancing scientific studies related to PSMC5-related neurodevelopmental disorders.

The variant was recently annotated in the ClinVar database by the OMIM submitter as a variant of uncertain significance (ID: VCV003899901.1; submission date: 25 May 2025). Notably, in the ClinVar database, a total of 38 PSMC5-related entries are currently reported, excluding our own submission. Among these, 23 are single-nucleotide variants (SNVs), 19 of which are classified as variants of uncertain significance (VUSs), most without a specified associated phenotype. One of these SNVs, the p.Met80Val variant (VCV003377264.1), has been described in association with a neurodevelopmental disorder, while four variants are classified as benign. Additionally, two small deletions are listed, both classified as VUSs; notably, the p.Lys393del variant (VCV003600378.1) has been linked to a “PSMC5-related neurodevelopmental disorder”. The remaining 13 entries correspond to copy number variants (CNVs), for which pathogenicity classifications and associated phenotypes are largely undefined.

To date, this variant has not been reported in either the gnomAD or dbSNP databases. Notably, the same p.Pro320Arg variant reported in this study was also identified in six individuals with neurodevelopmental disorders [12]. Two of these cases are documented in the DECIPHER database as de novo variants in patients with IDs 306096 and 307251. Patient 306096 presented with delayed speech and language development, joint hypermobility, and motor delay. Patient 307251 exhibited abnormalities of the proximal phalanx of the thumb, deeply set eyes, global developmental delay, a prominent forehead, a thin vermilion border, a wide mouth, and a broad nose. A phenotypic comparison between the individuals examined in this study and those previously reported is presented in Table S1 and was adapted from a previous study [12]. Based on these previously reported patients in the DECIPHER database, the *PSMC5* gene was recently included in the Simons Foundation Autism Research Initiative (SFARI) Gene database (Q1 2025 Release Notes), with a score of 3, indicating relatively weak but emerging evidence for its association with autism spectrum disorder (ASD).

**Figure 4:**
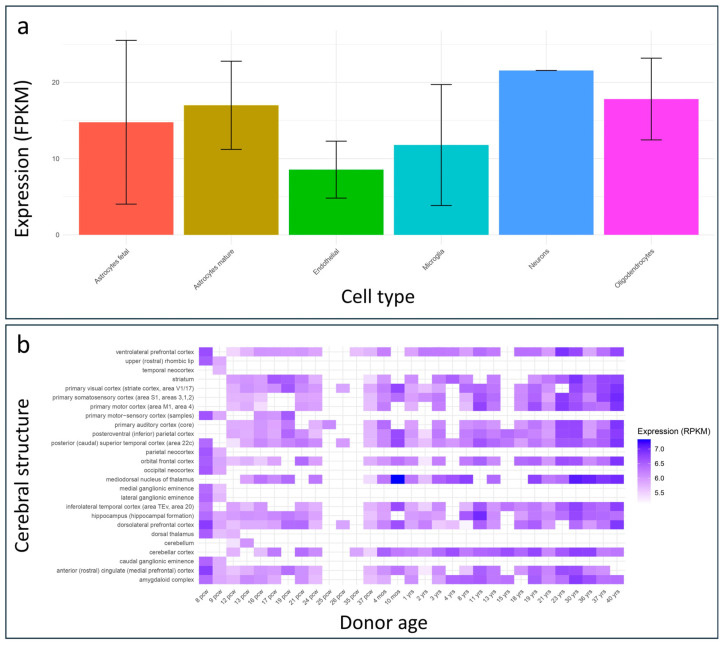
Brain expression of *PSMC5* across human brain structures. (**a**) Barplot illustrating *PSMC5* expression levels, measured in fragments per kilobase of transcript per million mapped reads (FPKM), across various human brain cell types. Data were retrieved from the BrainRNA-Seq database. (**b**) Heatmap representing developmental expression patterns of *PSMC5*, measured in reads per kilobase of transcript per million mapped reads (RPKM), across multiple human brain regions at different developmental stages. Data were obtained from the BrainSpan database.

According to the BrainRNA-Seq database, the *PSMC5* gene is highly expressed in the human brain, with particularly elevated expression in neurons (Figure 4a).

Complementary data from the BrainSpan developmental transcriptomic database indicate that *PSMC5* maintains high expression levels across various cerebral structures throughout development and adulthood (Figure 4b). Notably, the highest expression levels were observed in the mediodorsal nucleus of the thalamus at 10 months, 36 years, and 40 years of age. This brain region has been consistently implicated in neurodevelopmental disorders and is known to play a critical role in cognitive function [13,14].

The mutation is located at residue 320, within the 186–406 amino acid region of PSMC5, which has been described in mice as mediating the interaction with PRPF19 [15]. PRPF19 is a subunit of the spliceosome complex involved in the mRNA splicing process. It exhibits identical protein binding and ubiquitin ligase activities. Importantly, PRPF19 and other spliceosome components have been associated with neurodevelopmental disorders [16]. This variant has been proposed as a potential mutational hotspot—a hypothesis with which we concur. Functional in vitro studies investigating both PSMC5 insufficiency and the Pro320Arg mutation revealed that they impair proteasome activity and trigger apoptosis. Specifically, the Pro320Arg mutation disrupts proteasome function by altering the interaction between the 19S regulatory particle and the 20S core particle, as demonstrated by in vitro assays [12].

**Figure 5:**
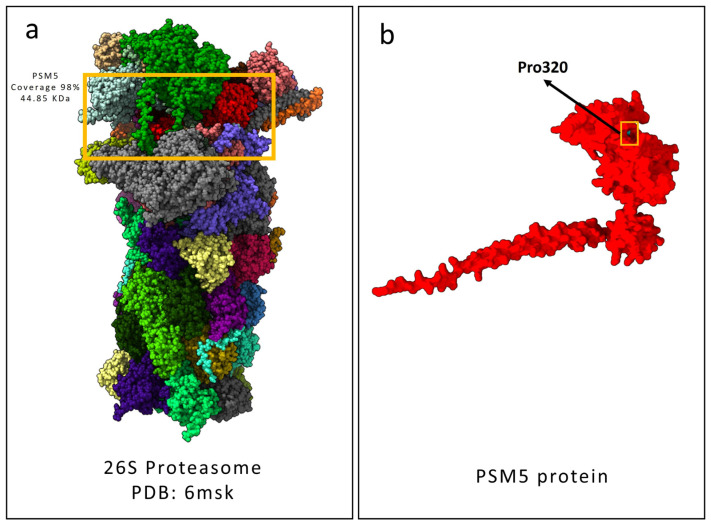
Structural depiction of the human 26S proteasome complex with a focus on the PSMC5 subunit. (**a**) Graphical representation of the 26S proteasome structure (PDB ID: 6MSK) deposited in the Protein Data Bank, with the PSMC5 subunit highlighted in red. (**b**) The predicted wild-type PSMC5 protein structure generated using the AlphaFold3 server is shown. Wild-type Pro320 is indicated by both the yellow square and the black arrow.

According to the Complex Portal entry for the 26S proteasome complex (ID: CPX-5993), six curated structural models of this protein machinery are available. For our analysis, we selected the structure with a PDB ID of 6msk (DOI: 10.2210/pdb6msk/pdb), which provides high-resolution structural information and the highest coverage. However, it is important to note that the Complex Portal does not explicitly specify the amino acid residues of PSMC5 involved in interactions with other proteasome subunits. Figure 5 shows the 26S PBD 6msk model compared with our best predicted model of PSMC5 generated using AlphaFold3.

A potential limitation of this study lies in the difficulty of accurately predicting the structural and functional consequences of the Pro320Arg mutation within the PSMC5 protein on the entire 26S proteasome complex. In fact, it would be highly interesting to perform a structural prediction of the entire 26S proteasome complex using AlphaFold-Multimer, including the PSMC5 protein carrying the variant identified in our study. However, due to the large number of polypeptide subunits that make up the 26S proteasome (over 10,000 amino acids in total), such an analysis would require significant computational resources, both in terms of processing power and RAM capacity. For this reason, we plan to postpone this analysis to a later stage, with the aim of estimating the mutation’s potential impact on the folding and architecture of the full proteasome complex. An alternative approach could involve the modeling of selected subcomplexes that include the mutated PSMC5 protein; however, this strategy may not provide a reliable estimation of the global structural impact. We anticipate that future advances in more powerful and cost-effective predictive algorithms, as well as greater access to high-performance computing infrastructures, will help overcome these current limitations.

It is also important to highlight that the patient examined in this study presented with thyroid abnormalities. As detailed in the clinical documentation, a thyroid ultrasound revealed a significantly enlarged left lobe with a hypoechoic and heterogeneous appearance, resembling a pseudonodular pattern. Additionally, the patient’s mother reported episodes of swallowing difficulty. Notably, both the mother and maternal grandmother had a history of thyroid disorders—thyroiditis and nodular goiter, respectively. We hypothesize that the de novo variant identified in the *PSMC5* gene may contribute to the development or worsening of thyroid abnormalities in the patient. This assumption is supported by previous findings indicating that the encoded protein subunit may be involved in transcriptional regulation, having been shown to interact with both the thyroid hormone receptor and retinoid X receptor-alpha [10]. Further studies are warranted to explore this potential interaction and to clarify the role of PSMC5 in thyroid hormone secretion and metabolism. It is worth noting that thyroid abnormalities were not reported in the six previously described individuals carrying the same genetic variant. Nonetheless, variants in other genes encoding subunits of the 26S proteasome—such as *PSMA1*, *PSMA3*, *PSMD3*, and *PSMD2*—have been associated with thyroid dysfunction [17]. It is worth mentioning that WES analysis identified as secondary finding the variant c.2200C>T (p.Arg734Trp) in the *FGFR1* gene in both the proband and the mother. This variant is documented in ClinVar (accessions: RCV001871469.5, RCV002482669.1, and RCV004699529.1) and classified as having uncertain significance. According to the OMIM database, FGFR1 is associated with the autosomal dominant disorders Hartsfield syndrome (#615465), hypogonadotropic hypogonadism (#147950), Jackson–Weiss syndrome (MIM #123150), and Pfeiffer syndrome (MIM #101600), exhibiting an incomplete penetrance. Although the thyroid hormone T3 modulates FGFR1 signaling [18,19,20], no explicit genotype-phenotype correlation links this variant to thyroid dysfunction. Thus, we cannot conclusively attribute the observed thyroid abnormalities to this variant.

This study aims to deepen the understanding of the *PSMC5* gene by expanding the spectrum of associated phenotypes and highlighting its previously suggested potential as a therapeutic target. Although functional studies are essential to confirm the association of the *PSMC5* gene and to validate the variant we identified as a potential mutational hotspot, we have submitted this variant to ClinVar (SCV006083352), classifying it as pathogenic and contributing to its first annotation with this classification in the database.

## 4. Materials and Methods

### 4.1. Library Preparation and WES Analysis

Genomic DNA was extracted from the peripheral blood leukocytes of the patient and both parents following a previously described procedure [21]. Library preparation for trio analysis and exome capture was performed using the Agilent SureSelect V7 Kit (Santa Clara, CA, USA), according to the manufacturer’s protocol. Sequencing was carried out using the Illumina HiSeq 3000 platform (San Diego, CA, USA), achieving coverage of at least 20× for 97% of the targeted regions. Variant filtering was performed based on (i) presumed inheritance patterns—recessive, de novo, or X-linked—and (ii) a minor allele frequency (MAF) below 1%, referencing population databases including 1000 Genomes, ESP6500, ExAC, and GnomAD. The human reference genome assembly HG38 was used for variant alignment and analysis. All identified variants were subsequently validated by conventional Sanger sequencing, performed with the BigDye™ Terminator v1.1 Cycle Sequencing Kit (Life Technologies, Carlsbad, CA, USA) on the SeqStudio Genetic Analyzer (Thermo Fisher Scientific, Waltham, MA, USA).

### 4.2. Data Analysis

**Figure 6:**
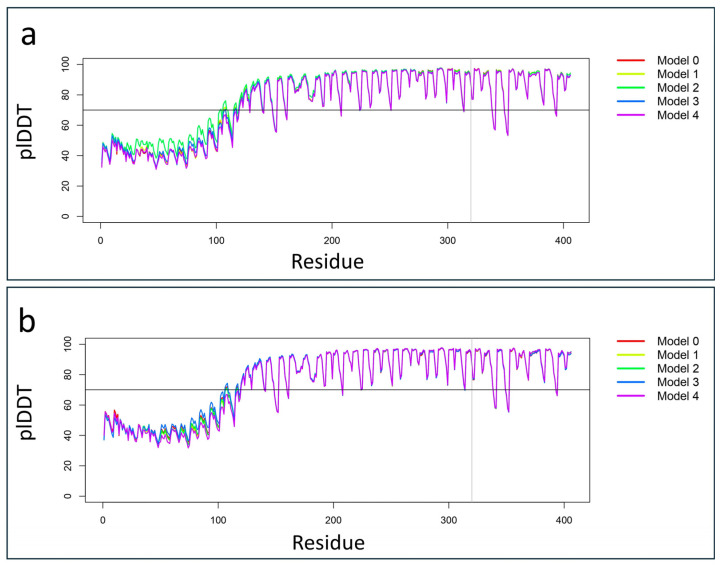
Line plots illustrate the variations in predicted local distance difference test (plDDT) scores across the five AlphaFold3 models for both the wild-type and p.Pro320Arg-mutated PSMC5 proteins. (**a**) The plot shows plDDT values for the wild-type PSMC5 predictions, highlighting “Model 3” as the best-performing model, with an average plDDT score of 81.125. (**b**) Similarly, the plot displays plDDT variations for the mutated p.Pro320Arg PSMC5 protein, where “Model 3” also emerged as the best model, with an average plDDT score of 80.396.

All the common variants, nonexonic polymorphisms, were excluded, keeping polymorphisms with a minor allele frequency (MAF) of <1% in the following public databases: gnomAD Exomes v.3.1.2, 1000 Genome Project, and Exome Sequencing Project (accessed on 23 May 2025). Pathogenic variants were searched against the Human Gene Mutation Database (HGMD Professional 2025.1, https://www.hgmd.cf.ac.uk/ac/introduction.php) (accessed on 23 May 2025). Franklin by QIAGEN was used for filtering and prioritizing the genetic variants from the vcf file. The identified variant was classified using the “American College of Medical Genetics” (ACMG) guidelines and criteria [22]. Multiple in silico analysis was performed using the VarSome platform [23]. The BrainRNAseq database (https://brainrnaseq.org/) (accessed on 23 May 2025) was used to obtain the expression of the *PSMC5* gene from different brain cells. Furthermore, the BrainSpan database (https://www.brainspan.org/) (accessed on 23 May 2025) was used for retrieving the transcriptomic data of cortical and subcortical structures across the full course of human brain development. Complex Portal (https://www.ebi.ac.uk/complexportal/home) (accessed on 23 May 2025) was used for studying the PSMC5 interactions within the proteasome 26 and for retrieving the accurate Protein Data Bank (PDB) structure of the P26 structure. Specifically, the structure considered was the 6msk structure (doi: 10.2210/pdb6msk/pdb). The structures of the wild-type and mutated PSMC5 proteins were predicted using the AlphaFold3 server (https://alphafoldserver.com/) (accessed on 23 May 2025). The “best model” with the highest plDDT value was selected across the five models for each prediction (Figure 6).

These structures were visualized and modeled using UCSF ChimeraX software version 1.8.

## 5. Conclusions

In the present study, we identified the c.959C>G (p.Pro320Arg) variant in the *PSMC5* gene in a patient diagnosed with global developmental delay and mild intellectual disability. This study aims to expand the spectrum of phenotypes associated with *PSMC5* and with this specific genetic variant, which has already been described in six other individuals. We emphasize the need for functional studies to validate the impact of this variant on the human phenotype and on the structural and biological stability of the 26S proteasome complex, which is pivotal for numerous cellular processes. We believe that the findings of this study may support the inclusion of *PSMC5* among the genes associated with neurodevelopmental disorders with an associated MIM phenotype number. Although such studies are necessary to confirm the gene-disease association and to validate the identified variant as a potential mutational hotspot, we have already submitted this variant to ClinVar (SCV006083352), classifying it as pathogenic and contributing to its first annotation with this classification in the database.

## Figures and Tables

**Table 1:** Multiple in silico analyses of the de novo variant c.959C>G p.Pro320Arg in the *PSMC5* gene identified by whole-exome sequencing (WES) analysis.

Tool	Prediction	Score
BayesDel addAF	Pathogenic Strong	0.468
MetaRNN	Pathogenic Strong	0.977
BayesDel noAF	Pathogenic Moderate	0.434
MetaSVM	Pathogenic Moderate	0.928
REVEL	Pathogenic Moderate	0.917
MetaLR	Pathogenic Supporting	0.835
AlphaMissense	Pathogenic Strong	0.996
EIGEN	Pathogenic Strong	1.070
EIGEN PC	Pathogenic Moderate	0.965
FATHMM-XF	Pathogenic Moderate	0.963
Mutation assessor	Pathogenic Moderate	4.180
MutPred	Pathogenic Moderate	0.858
PrimateAI	Pathogenic Moderate	0.898
PROVEAN	Pathogenic Moderate	−8.170
FATHMM-MKL	Pathogenic Supporting	0.992
LIST-S2	Pathogenic Supporting	0.979
LRT	Pathogenic Supporting	0.000
M-CAP	Pathogenic Supporting	0.583
SIFT	Pathogenic Supporting	0.000
SIFT4G	Pathogenic Supporting	0.000
BLOSUM	Uncertain	−5.000
DANN	Uncertain	0.998
DEOGEN2	Uncertain	0.788
FATHMM	Uncertain	−1.650
MutationTaster	Pathogenic	1.000
MVP	Uncertain	0.896

**Table 2:** List of the 23 hydrogen bonds identified in the best predicted wild-type PSMC5 model that are absent in the mutated p.Pro320Arg variant.

Donor Residue	Donor Atom	Acceptor Residue	Acceptor Atom	Distance (Å)
Gly17	N	Gly14	O	3.49
Ser26	OG	Gln22	O	3.44
Ser26	OG	Tyr23	O	2.78
Gln40	N	Asn36	O	2.94
Arg49	NE	Asn50	OD1	3.51
Glu68	N	Leu65	O	3.35
Tyr72	OH	Asn118	OD1	3.40
Lys101	N	Asp100	OD1	2.70
Ser120	OG	Asp119	OD1	3.09
Tyr121	OH	Glu92	OE2	3.21
His171	ND1	Glu173	OE1	2.96
Glu173	N	Glu173	OE2	2.76
Arg232	NE	Ala228	O	3.35
Gly265	N	Ser263	O	2.78
Ser267	OG	Gly264	O	2.92
Arg297	NE	Asp299	OD1	3.56
Ser303	OG	Asp302	OD1	3.56
Leu306	N	Ser303	O	3.22
Arg325	NH1	Pro320	O	2.93
Asn343	N	Gln380	OE1	3.00
Ser355	OG	Glu358	OE1	2.89
Ser400	OG	Glu396	O	2.94
Lys403	N	Met399	O	3.16

**Table 3:** List of the 21 hydrogen bonds identified in the best predicted p.Pro320Arg-mutated PSMC5 model that are absent in the wild-type structure.

Donor Residue	Donor Atom	Acceptor Residue	Acceptor Atom	Distance (Å)
Glu13	N	Leu11	O	2.80
Ser18	OG	Lys15	O	3.07
Glu68	N	Gln64	O	3.22
Asn106	N	Asp104	OD1	3.06
Asn118	ND2	Gln69	OE1	3.31
Asn129	ND2	Glu240	OE1	2.65
Asp132	N	Glu233	OE2	3.35
Ser136	OG	Asp132	O	3.45
Val140	N	Leu137	O	3.52
Arg201	NE	Glu141	OE2	2.99
His241	ND1	Met237	O	3.06
Asp266	N	Ser263	O	2.90
Val269	N	Gly265	O	3.36
Phe283	N	Gln279	O	3.28
Arg320	NH2	Thr194	O	2.19
Ala324	N	Asn321	OD1	2.82
Arg325	NH1	Arg320	O	2.81
Arg375	NH1	His377	O	2.92
Asp394	N	Val390	O	3.43
Ser395	OG	Gln392	O	2.68
Ser400	OG	Lys397	O	2.81

## Data Availability

The data presented in this study are available upon request from the corresponding author.

## Supplementary Materials

The supporting information can be downloaded at https://www.mdpi.com/article/10.3390/ijms26136386/s1.

## Abbreviations

The following abbreviations are used in this manuscript:

ACMG	American College of Medical Genetics and Genomics
ASD	Autism spectrum disorder
IGV	Integrated genomics viewer
MAF	Minor allele frequency
NGS	Next-generation sequencing
SFARI	Simons Foundation Autism Research Initiative
VUS	Variant of uncertain significance
